# Laccase-catalyzed derivatization of 6-aminopenicillanic, 7-aminocephalosporanic and 7-aminodesacetoxycephalosporanic acid

**DOI:** 10.1186/s13568-020-01117-0

**Published:** 2020-10-02

**Authors:** Annett Mikolasch, Elke Hammer, Sabine Witt, Ulrike Lindequist

**Affiliations:** 1 Institute of Microbiology, University Greifswald, Felix-Hausdorff-Straße 8, Greifswald, 17489 Germany; 2 Interfaculty Institute for Genetics and Functional Genomics, University Greifswald, Felix-Hausdorff-Straße 8, Greifswald, 17489 Germany; 3 Biometec, Walther-Rathenau-Str. 49a, Greifswald, 17489 Germany; 4 Institute of Pharmacy University Greifswald, Friedrich-Ludwig-Jahn-Str. 17, Greifswald, 17487 Germany

**Keywords:** Laccase, Biotransformation, ß-lactam antibiotics, Antibacterial activity

## Abstract

*Trametes spec.* laccase (EC 1.10.3.2.) mediates the oxidative coupling of 6-aminopenicillanic, 7-aminocephalosporanic, and 7-aminodesacetoxycephalosporanic acid with 2,5-dihydroxybenzoic acid derivatives to form new penicillin and cephalosporin structures, respectively. The heteromolecular hybrid dimers are formed by nuclear amination of the *p*-hydroquinones with the primary amines and inhibited *in vitro* the growth of *Staphylococcus* species, including some multidrug-resistant strains.

## Key points

Fungal laccase catalyzes the coupling of 2,5-dihydroxybenzoic acid derivatives with the core units of penicillins and cephalosporins.

In contrast to the inactive educts the coupling products possess weak to moderate antibacterial activity.

Chinoid substructures impact antimicrobial spectrum and activity.

## Introduction

The laccase-mediated reaction is a well-described method to synthesize novel antibiotics by enzymatic catalysis (Agematu et al. 1993; Anyanwutaku et al. 1994; Mikolasch et al. 2016; Zhang et al. 2020). These reactions enable the use of low-cost processes, mild reaction conditions-aqueous solvent systems, normal pressure, room temperature - to synthesize novel antibiotics. A further advantage is the specificity of the laccase-initiated reaction, which catalyse the amination and thiolation of *para*- and *ortho*-dihydroxylated aromatic compounds (Abdel-Mohsen et al. 2014; Manda et al. 2005; Niedermeyer et al. 2005; Patel and Gupte 2018; Schlippert et al. 2016; Wellington et al. 2013). Further examples of useful modifications are laccase-mediated reactions in which two antibiotics containing a phenol moiety are combined (Agematu et al. 1993), or where a phenolic compound is added into an antibiotic containing a phenolic moiety (Anyanwutaku et al. 1994), or the synthesis or transformation of heterocyclic compounds (Mikolasch and Schauer 2003; Saadati et al. 2018; Schäfer et al. 2001; Youssef et al. 2020).

Recently we reviewed the laccase-mediated synthesis of novel antibiotics (Mikolasch 2019). Several of the reported products inhibited the growth of gram positive bacterial strains and protected mice against severe disease after infection with *Staphylococcus aureus* (Mikolasch et al. 2008, 2012, 2016). In all of our work so far, we have focused on the transformation of existing approved ß-lactam antibiotics. However, many bacteria, including clinically significant *Staphylococcus* and *Streptococcus* species, have developed resistance to these antibiotics so that novel antibiotic structures are urgently needed to replace them (Blinder et al. 2019; Fisher et al. 2005; Helfand and Bonomo 2005; Mbaye et al. 2019; Shimizu et al. 2001).

Therefore, in this study we used the core structural elements of ß-lactam antibiotics, the 6-aminopenicillanic, 7-aminocephalosporanic, and 7-aminodesacetoxycephalosporanic acid, as coupling partners for laccase-mediated reactions. The aim was to change the C-6 and C-7 substituent respectively, as has been previously achieved (Dabernat et al. 2004; Lam et al. 2015; Lin et al. 2003; Lopez et al. 2004; Potron et al. 2013; Springer et al. 2003; Stachulski 1985), but by using a novel synthesis approach.

We have employed laccase C from *Trametes spec.* to achieve derivatization of the core structures of ß-lactam antibiotics and to couple them with 2,5-dihydroxy-N-(2-hydroxyethyl)benzamide and 2,5-dihydroxybenzoic acid methyl ester. These 2,5-dihydroxybenzoic acid derivatives are structurally related to the ganomycins, another class of antibacterial compounds (Mothana et al. 2000). The novel heteromolecular hybrid dimers were characterized by LC/MS and NMR data and the antimicrobial activity assayed in an agar diffusion and a cytotoxicity test.

## Materials and methods

### Enzyme

Extracellular laccase C of *Trametes spec.* (EC 1.10.3.2) was obtained from ASA Spezialenzyme (Wolfenbüttel, Germany) and used in an activity of 800 nmol⋅mL^− 1^⋅min^− 1^ (substrate: 2,2’-amino-bis-3-ethylbenzthiazoline-6-sulfonic acid).

### Substrates and conditions of biotransformation

6-Aminopenicillanic, 7-aminocephalosporanic and 7-aminodesacetoxycephalosporanic acid (2 mM) were dissolved in 600 ml sodium acetate buffer, 20 mM pH 5.6. After addition of laccase C (activity 800 nmol⋅mL^− 1^⋅min^− 1^), 2,5-dihydroxybenzoic acid derivatives− 2,5-dihydroxy-N-(2-hydroxyethyl)benzamide or 2,5-dihydroxybenzoic acid methyl ester – were added (60 ml of a 20 mM solution in sodium acetate buffer, pH 5.6). The reaction mixture was incubated for 3 h at room temperature (RT) with agitation at 400 rpm.

Chemicals were purchased from commercial suppliers: 2,5-dihydroxybenzoic acid methyl ester, 6-aminopenicillanic, 7-aminocephalosporanic and 7-aminodesacetoxycephalosporanic acid from Sigma-Aldich (Germany) and 2,5-dihydroxy-N-(2-hydroxyethyl)benzamide from Midori Kagaku Co (Japan).

### Analytical high-performance liquid chromatography (HPLC) and general procedure for isolation of biotransformation products

For routine analysis, samples of the incubation mixture were analyzed by HPLC and use of substrates and product synthesis recorded by a UV detector. The isolation of the coupling products was performed by solid phase extraction. Methods were described in detail by Mikolasch et al. (2006).

### Characterization of biotransformation products

Products were analyzed by mass spectrometry (LC/MS with API-ES in negative and positive modes) and FT-ICR MS high-resolution mass spectrometry experiments as described by Manda et al. (2006). The nuclear magnetic resonance (NMR) spectra were obtained at 300 MHz (^1^H) in acetonitrile-d_3_.

#### 7-[[2-(2-Hydroxyethylcarbamoyl)-3,6-dioxocyclohexa-1,4-dien-1-yl]amino]-penicillanic acid 3a 

Yield 66%, ^1^H NMR δ 1.50 (s, 3H, H-9 or H-10), 1.56 (s, 3H, H-9 or H-10), 3.39 (m, ^3^J = 5.5 Hz, 2H, H-9´), 3.59 (t, ^3^J = 5.5 Hz, 2H, H-10´), 4.44 (s, 1H, H-3), 5.64 (dd, ^3^J = 4.0 Hz,1H, H-5), 5.72 (d, ^3^J = 4.0 Hz, 1H, H-6), 6.63 (d, ^3^J = 10.3 Hz, 1H, H-4´), 6.71 (d, ^3^J = 10.3 Hz, 1H, H-5´), 9.67 (s(broad), 1H, H-8´), 12.99 (s(broad), 1H, H-11). LC/MS *m*/*z* 408.0 ([M-H]^−^ API-ES neg. mode), 410.3 ([M + H]^+^ API-ES pos. mode), HRMS (C_17_H_19_N_3_O_7_S): calcd: 409.09437, found: 409.09441.

#### 7-[(2-Methoxycarbonyl-3,6-dioxocyclohexa-1,4-dien-1-yl)amino]-penicillanic acid 3b

Yield 66%, ^1^H NMR δ 1.52 (s, 3H, H-9 or H-10), 1.55 (s, 3H, H-9 or H-10), 3.80 (s, 3H, H-8´), 3.95 (s, 1H, H-3), 5.52 (m, ^3^J = 4.0 Hz,1H, H-5), 5.63 (d, ^3^J = 4.0 Hz, 1H, H-6), 6.63 (d, ^3^J = 10.1 Hz, 1H, H-4´), 6.71 (d, ^3^J = 10.1 Hz, 1H, H-5´), 11.59 (s(broad), 1H, H-11), LC/MS *m*/*z* 379.2 ([M-H]^−^ API-ES neg. mode), 381.3 ([M + H]^+^ API-ES pos. mode), HRMS (C_16_H_16_N_2_O_7_S): calcd: 380.06782, found: 380.06789.

#### 3-(Acetoxymethyl)-7-[[2-(2-hydroxyethylcarbamoyl)-3,6-dioxocyclohexa-1,4-dien-1-yl]amino]-cephalosporanic acid 3c

Yield 68%, ^1^H NMR δ 2.01 (s, 3H, H-12), 3.41 (d, ^2^J = 18.3 Hz, 1H, H-2), 3.40 (m, ^3^J = 5.5 Hz, 2H, H-9´), 3.59 (t, ^3^J = 5.5 Hz, 2H, H-10´), 3.64 (d, ^2^J = 18.3 Hz, 1H, H-2), 4.77 (d, ^2^J = 13.2 Hz, 1H, H-10), 5.05 (d, ^2^J = 13.2 Hz, 1H, H-10), 5.26 (d, ^3^J = 4.7 Hz,1H, H-6), 5.79 (m, ^3^J = 4.7 Hz, 1H, H-7), 6.66 (d, ^3^J = 10.3 Hz, 1H, H-4´), 6.72 (d, ^3^J = 10.3 Hz, 1H, H-5´), 9.64 (s(broad), 1H, H-8´), 12.96 (s(broad), 1H, H-13). LC/MS *m*/*z* 464.0 ([M-H]^−^ API-ES neg. mode), 466.2 ([M + H]^+^, 488.1 [M + Na]^+^ API-ES pos. mode), HRMS (C_19_H_19_N_3_O_9_S): calcd: 465.08420, found: 465.08431.

#### 3-(Acetoxymethyl)-7-[(2-methoxycarbonyl-3,6-dioxocyclohexa-1,4-dien-1-yl)amino]-cephalosporanic acid 3d

Yield 63%, ^1^H NMR δ 2.00 (s, 3H, H-12), 3.30 (d, ^2^J = 18.0 Hz, 1H, H-2), 3.59 (d, ^2^J = 18.0 Hz, 1H, H-2), 3.81 (s, 3H, H-8´), 4.81 (d, ^2^J = 12.9 Hz, 1H, H-10), 5.05 (d, ^2^J = 12.9 Hz, 1H, H-10), 5.13 (d, ^3^J = 4.1 Hz,1H, H-6), 5.47 (m, ^3^J = 4.1 Hz, 1H, H-7), 6.64 (d, ^3^J = 10.2 Hz, 1H, H-4´), 6.72 (d, ^3^J = 10.2 Hz, 1H, H-5´), 11.65 (s(broad), 1H, H-13). LC/MS *m*/*z* 434.9 ([M-H]^−^ API-ES neg. mode), 437.2 ([M + H]^+^ API-ES pos. mode), HRMS (C_18_H_16_N_2_O_9_S): calcd: 436.05765, found: 436.05778.

#### 7-[[2-(2-Hydroxyethylcarbamoyl)-3,6-dioxocyclohexa-1,4-dien-1-yl]amino]-desacetoxy-cephalosporanic acid 3e

Yield 73%, Synthesis and isolation as described above. Dark red solid. ^1^H NMR δ 2.09 (s, 3H, H-10), 3.24 (d, ^2^J = 18.1 Hz, 1H, H-2), 3.40 (m, ^3^J = 5.5 Hz, 2H, H-9´), 3.59 (d, ^2^J = 18.1 Hz, 1H, H-2), 3.60 (t, ^3^J = 5.5 Hz, 2H, H-10´), 5.21 (d, ^3^J = 4.6 Hz,1H, H-6), 5.72 (dd, ^3^J = 4.6 Hz, J = 5.6 Hz, 1H, H-7), 6.66 (d, ^3^J = 10.2 Hz, 1H, H-4´), 6.72 (d, ^3^J = 10.1 Hz, 1H, H-5´), 9.65 (s(broad), 1H, H-8´), 12.95 (s(broad), 1H, H-11). LC/MS *m*/*z* 406.0 ([M-H]^−^ API-ES neg. mode), 408.2 ([M + H]^+^, 430.1 [M + Na]^+^ API-ES pos. mode), HRMS (C_17_H_17_N_3_O_7_S): calcd: 407.07872, found: 407.07881.

#### 7-[(2-Methoxycarbonyl-3,6-dioxocyclohexa-1,4-dien-1-yl)amino]-desacetoxy-cephalosporanic acid 3 f

Yield 71%, ^1^H NMR δ 2.07 (s, 3H, H-10), 3.22 (d, ^2^J = 17.9 Hz, 1H, H-2), 3.53 (d, ^2^J = 17.9 Hz, 1H, H-2), 3.81 (s, 3H, H-8´), 5.11 (d, ^3^J = 3.9 Hz,1H, H-6), 5.46 (m, ^3^J = 3.9 Hz, 1H, H-7), 6.63 (d, ^3^J = 10.2 Hz, 1H, H-4´), 6.72 (d, ^3^J = 10.2 Hz, 1H, H-5´), 10.15 (s(broad), 1H, H-11). LC/MS *m*/*z* 377.2 ([M-H]^−^ API-ES neg. mode), 379.2 ([M + H]^+^ API-ES pos. mode), HRMS (C_16_H_14_N_2_O_7_S): calcd: 378.05217, found: 378.05225.

### Determination of antibacterial activity

An agar diffusion method described previously was used to determine antibacterial activity in the range from 19 to 190 nmol (Mikolasch et al. 2007). The following bacterial strains were used: *Staphylococcus aureus* ATCC 6538, *S. aureus* Norddeutscher Epidemiestamm and the multidrug resistant strains isolated from patients *S. aureus* 315 and *S. epidermidis* 99,847.

### Cytotoxic activity

Cytotoxicity was determined by a neutral red uptake assay using FL-cells, a human amniotic epithelial cell line, as reported before (Mikolasch et al. 2007).

## Results

### Biotransformation of 6-aminopenicillanic acid by laccase

**Fig. 1:**
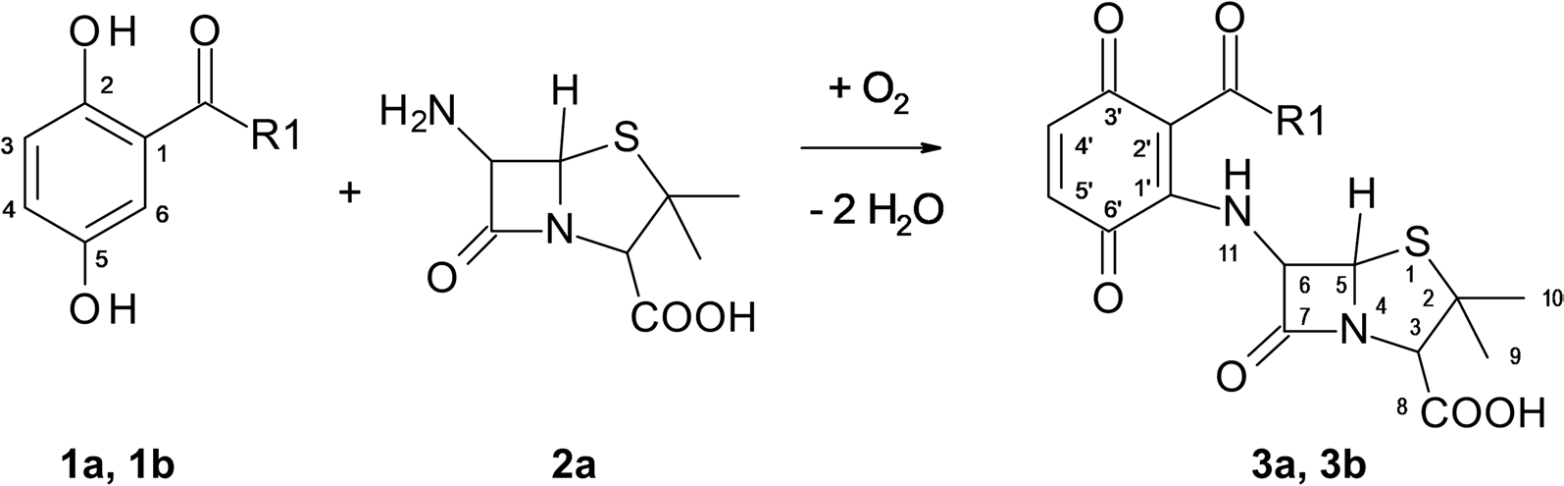
2,5-Dihydroxybenzoic acid derivatives (**1a** R1 = NH(CH_2_)_2_OH and **1b** R1 = OOCH_3_), 6-aminopenicillanic acid (**2a**) and the products **3a** (R1 = NH(CH_2_)_2_OH) and **3b (**R1 = OOCH_3_)

Laccase-mediated reactions of 2,5-dihydroxy-N-(2-hydroxyethyl)benzamide **1a** and 2,5-dihydroxybenzoic acid methyl ester **1b** with 6-aminopenicillanic acid **2a** both resulted in one heteromolecular hybrid dimer each, **3a** and **3b** (Fig. 1).

After separation of **3a** and **3b** by solid phase extraction, LC/MS in negative and positive mode and HRMS analyses revealed molecular masses of the products attributed to the formation of heteromolecular hybrid dimers consisting of a structural part of a 2,5-dihydroxybenzoic acid derivative (**1a** or **1b**) coupled to 6-aminopenicillanic acid **2a** accompanied by the loss of four hydrogen atoms. These couplings were confirmed by the presence of characteristic signals for **1a** or **1b** and for **2a** in the ^1^H NMR spectra of **3a** and **3b**. The number of CH proton signals of the 2,5-dihydroxybenzoic acid derivatives changed from three in the substrate, to two signals in the products. The multiplicity of the proton signals H-4’ and H-5’ indicated an additional substituent at the C-1’ atom and the loss of a proton. The chemical shift to lower field of the H-4’ and H-5’’ signals confirmed the presence of an electron-withdrawing group. Signals for phenolic hydroxyl groups could not be measured, but instead additional amine protons were detected. All analytical data confirmed the oxidation of the *p*-hydroquinone structure of **1a** and **1b** to a quinone.

**Fig. 2:**
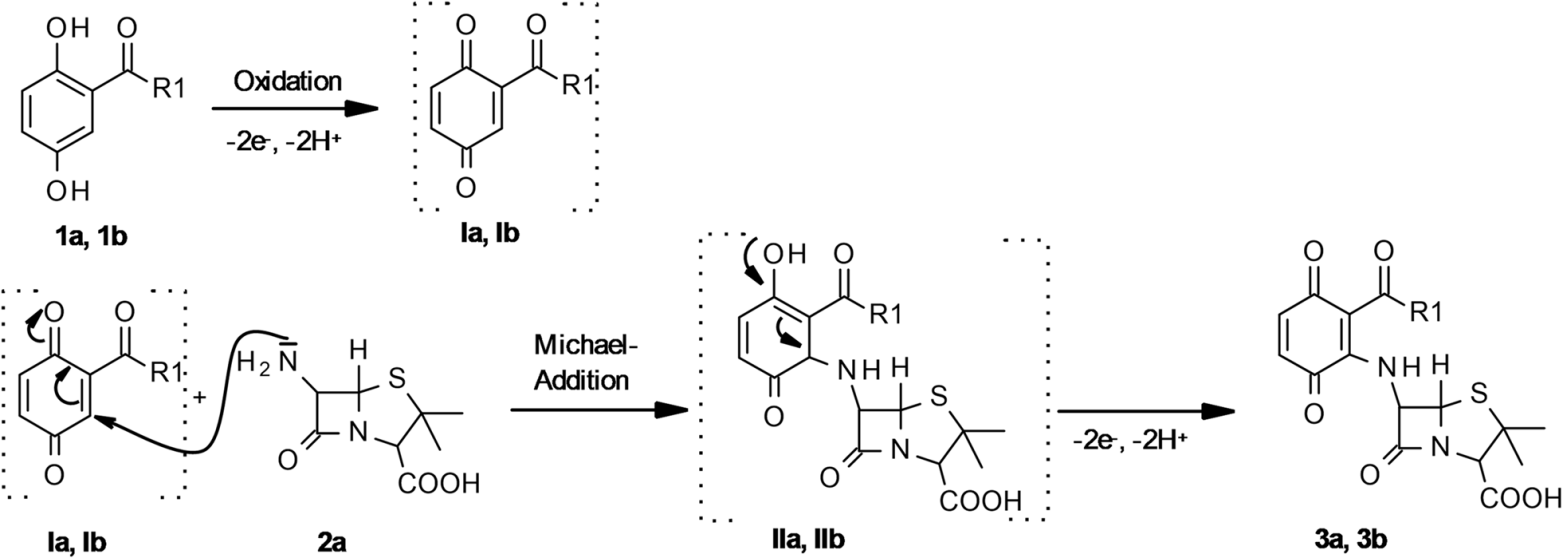
Reaction mechanism of 2,5-dihydroxybenzoic acid derivatives (**1a**, **Ia**, **IIa**, **3a** R1 = NH(CH_2_)_2_OH and **1b, Ib**, **IIb**, **3b** R1 = COOCH_3_) and 6-aminopenicillanic acid (**2a**)

The heteromolecular hybrid dimers **3a** and **3b** are formed by nuclear amination of the *p*-hydroquinones **1a** or **1b** with the primary amino group of **2a** (Fig. 2).

### Biotransformation of 7-aminocephalosporanic and 7-aminodesacetoxycephalosporanic acid by laccase

**Fig. 3:**
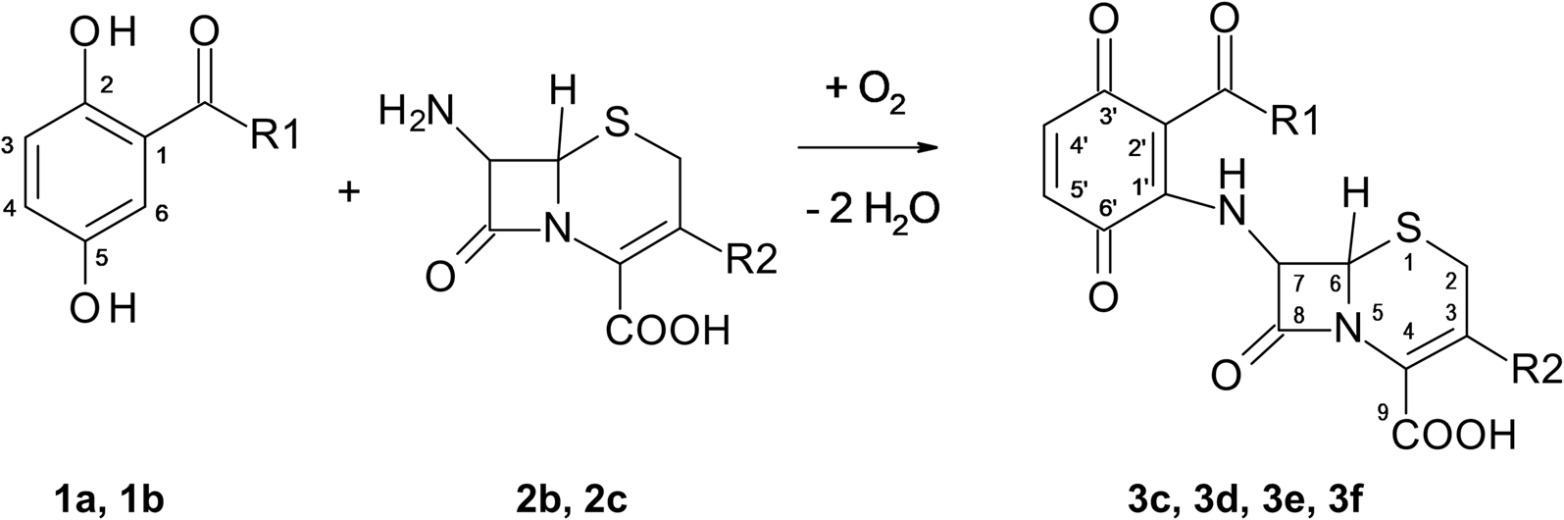
2,5-Dihydroxybenzoic acid derivatives (**1a** R1 = NH(CH_2_)_2_OH and **1b** R1 = COOCH_3_), 7-aminocephalosporanic acid (**2b** R2 = CH_2_OCOCH_3_), 7-aminodesacetoxy-cephalosporanic acid (**2c** R2 = CH_3_) and the products **3c** (R1 = NH(CH_2_)_2_OH, R2 = CH_2_OCOCH_3_), **3d** (R1 = COOCH_3_, R2 = CH_2_OCOCH_3_), **3e** (R1 = NH(CH_2_)_2_OH, R2 = CH_3_) and **3f** (R1 = COOCH_3_, R2 = CH_3_)

7-Aminocephalosporanic **2b** and 7-aminodesacetoxycephalosporanic acid **2c** reacted in the same way as 6-aminopenicillanic acid **2a** with 2,5-dihydroxy-N-(2-hydroxyethyl)benzamide **1a** and 2,5-dihydroxybenzoic acid methyl ester **1b** under the presence of laccase. A heteromolecular hybrid dimer, **3c**, **3d**, **3e** or **3f**, was recoved from each reaction (Fig. 3).

The extraction by solid phase and the structural analysis by LC/MS, HRMS and NMR of products **3c** to **3f** gave comparable results to the products **3a** and **3b**. The reactions are expected to follow the same mechanism.

### Biological activity of the biotransformation products

The products **3a** to **3f** caused low to moderate growth inhibition of *S. aureus* and *S*. *epidermidis* strains, among them multidrug resistant staphylococci (Table 1). In contrast, the educts **1a** and **1b** and **2a** to **2c** were not active against the strains tested. This indicates that by laccase-mediated reaction a product with antimicrobial activity can be produced from two initially inactive compounds. The previously described products **3A**, **3B** and **3C** (Mikolasch et al. 2006, 2007) were built from inactive 2,5-dihydroxybenzoic acid derivatives **1a** or **1b** and from the clinically relevant antibiotics amoxicillin or cefadroxil. The antibiotic activity of **3A**, **3B**, and **3C** was comparable to that of the educts amoxicillin and cefadroxil.

**Table 1:** Antimicrobial activity of products **3a** to **3f**, educts **2a** to **2c** and comparable data from previous research (educts **2A** to **2C** and products **3A** to **3C**)

Strain	Amount n [µmol]														
	**2a**			**3a**			**3b**			**2A (AM)**^c^			**3A (1a + AM)**^c^		
	0.019	0.1	0.19	0.019	0.1	0.19	0.019	0.1	0.19	0.019	0.1	0.19	0.019	0.1	0.19
*Staphylococcus aureus* ATCC 6538	r^a^	r	r	12^b^	18	22	r	24	30	36	42	44	26	30	34
*S. aureus* Norddeutscher Stamm	r	r	r	r	16	18	r	r	16	r	12	14	r	10	14
*S. aureus* 315	r	r	r	r	r	r	r	r	r	8	14	16	r	8	12
*S. epidermidis* 99,847	r	r	r	r	r	r	r	r	r	20	24	26	14	20	22

Strain	Amount n [µmol]														
	**2b**			**3c**			**3d**			**2B (CD)**^d^			**3B (1a + CD)**^d^		
	0.019	0.1	0.19	0.019	0.1	0.19	0.019	0.1	0.19	0.019	0.1	0.19	0.019	0.1	0.19
*S. aureus* ATCC 6538	r	r	r	r	14	18	r	r	10	32	38	40	20	28	30
*S. aureus* Norddeutscher Stamm	r	r	r	r	20	24	r	r	r	r	r	10	r	16	18
*S. aureus* 315	r	r	r	r	r	r	r	r	r	r	r	8	r	10	16
*S. epidermidis* 99,847	r	r	r	r	r	22	r	r	14	20	24	26	r	10	16

Strain	Amount n [µmol]														
	**2c**			**3e**			**3f**			**2B (CD)**^d^			**3C (1b + CD)**^d^		
	0.019	0.1	0.19	0.019	0.1	0.19	0.019	0.1	0.19	0.019	0.1	0.19	0.019	0.1	0.19
*S. aureus* ATCC 6538	r	r	r	r	18	22	r	14	30	32	38	40	18	26	30
*S. aureus* Norddeutscher Stamm	r	r	r	r	20	22	r	r	r	r	r	10	r	10	14
*S. aureus* 315	r	r	r	r	r	r	r	r	r	r	r	8	r	r	12
*S. epidermidis* 99,847	r	r	r	r	r	24	r	r	r	20	24	26	r	14	20

^a^ Resistant (no zone of inhibition)

^b^ Zones of inhibition (mm)

^c^ Educt **2A** and product **3A** are described previously (Mikolasch et al. 2006); *AM* amoxicillin

^d^ Educt **2B** and products **3B**, **3C** are described previously (Mikolasch et al. 2007); *CD* cefadroxil

Good product stability was demonstrated by HPLC measurement when the compounds were stored in solid form at 4 °C for several weeks. However, incubation of the compounds **3a** to **3f** in aqueous solutions at 30 °C resulted in decomposition within three hours. For this reason, the survey of the antimicrobial effects was restricted to agar diffusion tests.

**3a** to **3f** showed no cytotoxicity against FL cells in concentrations up to 100 µg/ml (data not shown).

## Discussion

With the use of laccase C from *Trametes spec.*, the laccase substrates **1a** and **1b** were consumed rapidly, resulting in high yields of the cross coupling product **3a** to **3 f.** Similar straightforward biotransformations of educts to products were described for hybrid dimer formation from the laccase substrates **1a** and **1b** (*para*-dihydroxylated) with aminopenicillins, aminocephalosporins, and aminocarbacephems (Mikolasch et al. 2006, 2007).

In contrast, divergent reaction kinetics were observed for hybrid dimer formation from syringic acid (monohydroxylated and *meta*-dimethoxylated) and 3,4-dichloroaniline (Tatsumi et al. 1994) or from 3,4-dihydroxyaromatic acid substructures (*ortho*-dihydroxylated) and ß-lactam antibiotics (Mikolasch et al. 2012). Therefore, the laccase-mediated reaction of **1a** and **1b** with 6-aminopenicillanic **2a**, 7-aminocephalosporanic **2b**, and 7-aminodesacetoxycephalosporanic acid **2c** again confirmed that *para*-dihydroxylated laccase substrates are preferable reaction partners in these syntheses in comparison to *ortho*-substituted compounds.

The structural analyses indicate that the products **3a** to **3f** are quinoids formed by Michael addition (Fig. 2). Despite this structural equivalence to the products described in other studies (3A to 3C, Table 1), there was only a comparatively low antibacterial activity against *Staphylococcus* strains (Table 1). Amoxicillin (**2A**, AM, Table 1), an α-amino-*p*-hydroxybenzyl penicillin with a structure similar to **3a** and **3b**, is a semisynthetic derivative of penicillin but with better absorption and higher concentrations in blood and in urine. It is recommended for treatment of acute otitis media (Handsfield et al. 1973; Zimmermann and Peterson 2006). Cefadroxil (**2B**, CD, Table 1), an α-amino-*p*-hydroxybenzyl cephalosporin with a structure similar to **3e** and **3f**, is a derivative of cephalosporin effective in Gram-positive and Gram-negative bacterial infections (Beauduy and Winston 2018; de Marco and Salgado 2017). Both AM and CD have a hydroxylated aromatic ring within the structure attached at the 6- or 7-position of the ß-lactam basic structure, while products **3a**, **3b**, **3e**, and **3f** contain a quinoid ring directly attached to these positions. These structural differences could result in a low shielding of the ß-lactam structure. The β-lactam structures are analogues of d-alanyl-d-alanine, the terminal amino acid residues on the precursor NAM/NAG-peptide subunits of the nascent peptidoglycan layer. The β-lactam nuclei irreversibly bind to the active site of bacterial transpeptidases involved in peptidoglycan synthesis. Therefore, they act as inhibitors of the cross-linkage between the linear peptidoglycan polymer chains and disrupt the cell wall synthesis (Fisher et al. 2005). If the bacteria produce β-lactamases, these enzymes cleave the β-lactam ring and reduce the antibiotic effect (Drawz and Bonomo 2010). In case of less well-shielded β-lactam nuclei, the antibiotics might be susceptible to bacterial degradation and thereby less active as documented for **3a** to **3 f.** However, these products were built from two inactive substances by laccase-mediation. In contrast, the previously described products **3A**, **3B** and **3C** produced from an antibiotic AM or CD and from an inactive 2,5-dihydroxybenzoic acid derivative only had comparable antibiotic activity to the educts AM or CD.

The structures of **3a** to **3f** include a *p*-quinoid unit. Several compounds with *p*-quinone clusters are important chemotherapeutics with cytostatic activity (Ikushima et al. 1980; Nweze et al. 2020; Pachatouridis et al. 2002; Whitesell et al. 1992). Therefore, it is remarkable that **3a** to **3f** did not show a cytotoxic effect on FL cells, though effects on eukaryotic cells can differ, as reported for geldanamycin, a *p*-benzoquinone antitumor antibiotic (Whitesell et al. 1992). No cytotoxic effect on FL cells was also observed for the previously described products **3A**, **3B**, and **3C** that also include a *p*-quinoid unit. (Mikolasch et al. 2006, 2007).

With the present study we demonstrate that two inactive educts can be linked together by laccase-mediation to form an active heteromolecular product at high yield. However, the impact of product substructures on antimicrobial activity and bacterial sensitivity have to be tested more intensively in order to exploit the whole potential of the chinoid products **3a** to **3f** as well as **3A**, **3B**, and **3C**.
